# Investigating the Improvement of Decoding Abilities and Working Memory in Children with Incremental or Entity Personal Conceptions of Intelligence: Two Case Reports

**DOI:** 10.3389/fpsyg.2015.01939

**Published:** 2016-01-05

**Authors:** Marianna Alesi, Gaetano Rappo, Annamaria Pepi

**Affiliations:** Dipartimento di Scienze Psicologiche, Pedagogiche e della Formazione, Università degli Studi di PalermoPalermo, Italy

**Keywords:** personal conceptions of intelligence, working memory, learning disabilities, dyslexia, intervention program, children, case report

## Abstract

One of the most significant current discussions has led to the hypothesis that domain-specific training programs alone are not enough to improve reading achievement or working memory abilities. Incremental or Entity personal conceptions of intelligence may be assumed to be an important prognostic factor to overcome domain-specific deficits. Specifically, incremental students tend to be more oriented toward change and autonomy and are able to adopt more efficacious strategies. This study aims at examining the effect of personal conceptions of intelligence to strengthen the efficacy of a multidimensional intervention program in order to improve decoding abilities and working memory. Participants included two children (M age = 10 years) with developmental dyslexia and different conceptions of intelligence. The children were tested on a whole battery of reading and spelling tests commonly used in the assessment of reading disabilities in Italy. Afterwards, they were given a multimedia test to measure motivational factors such as conceptions of intelligence and achievement goals. The children took part in the T.I.R.D. Multimedia Training for the Rehabilitation of Dyslexia (Rappo and Pepi, 2010) reinforced by specific units to improve verbal working memory for 3 months. This training consisted of specific tasks to rehabilitate both visual and phonological strategies (sound blending, word segmentation, alliteration test and rhyme test, letter recognition, digraph recognition, trigraph recognition, and word recognition as samples of visual tasks) and verbal working memory (rapid words and non-words recognition). Posttest evaluations showed that the child holding the incremental theory of intelligence improved more than the child holding a static representation. On the whole this study highlights the importance of treatment programs in which both specificity of deficits and motivational factors are both taken into account. There is a need to plan multifaceted intervention programs based on a transverse approach, considering both cognitive and motivational factors.

## Introduction

One of the most significant current discussions has led to the hypothesis that domain-specific training programs alone are not enough to improve decoding abilities or working memory (Ho and Guthrie, 2013; Jaeggi et al., 2014). It is becoming increasingly evident that personal conceptions of intelligence play a key role as prognostic variables in the planning of training programs to rehabilitate reading and memory deficits. This is because of the way intelligence is conceived is assumed to sustain and maintain the readiness to recover own personal difficulties and to be oriented toward change and autonomy through training (Pepi et al., 2008).

In their original model, Dweck and colleagues hypothesized two theories or personal conceptions concerning the nature of intelligence and ability, namely incremental and entity theories (Dweck and Leggett, 1988; Faria, 1998; Dweck, 1999). In particular, incremental theorists conceive their intelligence as a resource which can be increased through personal engagement and effort. Consequently, they tend to choose learning goals which allow them to prefer challenging tasks and employ successful strategies in order to improve their abilities. Whilst, entity theorists perceive their ability as a unchangeable talent with which the person is endowed. Consequently, they are likely to prefer performance goals aimed at demonstrating their abilities and obtain positive evaluations from others (Dweck, 1999; Pepi et al., 2015).

Experimental research has shown a positive relationship between incremental personal conceptions of intelligence and school success (Stipek and Gralinski, 1996; Robins and Pals, 2002; Pepi et al., 2006). Moreover, intervention programs aimed at teaching incremental conceptions of intelligence were demonstrated to reduce achievement discrepancies; for example, students taught in incremental view, compared with control groups, were found to achieve higher grades in maths and science performance (Aronson et al., 2002; Good et al., 2003). Furthermore, personal conceptions of intelligence are predictive over time; in a longitudinal study Blackwell et al. (2007) followed 7th graders over 2 years and found that their mindset at the beginning of junior high school was associated to their trajectories of maths achievement. Students holding an incremental mindset had higher gains in maths grades compared to their peers holding an entity mindset.

With regard to reading training programs, research suggests that domain-specific training programs alone are not sufficient to improve reading abilities. Considering the mutual enhancement among a student's cognitive and emotional-motivational attributes in the reading abilities, an integrated program was demonstrated to be more efficacious (Cox and Guthrie, 2001; Guthrie et al., 2007; Villavicencio and Bernardo, 2013). This is because a training program tapping simultaneously cognitive and motivational abilities is assumed to sustain more effectively the maintenance and generalization of the obtained gains (Pepi et al., 2000). Pepi et al. (2008) compared improvements in reading accuracy and speed abilities of pupils with incremental and entity personal conceptions of intelligence following reading decoding treatment. The results showed that both groups improved their abilities in reading decoding but incremental theorists showed larger gain percentage scores than static ones. Moreover, in the incremental group, gains on reading accuracy and speed were found to be more relevant at Follow-up after 3 months. Previously obtained results were corroborated (Pepi et al., 2004) which revealed more consistent improvements in reading comprehension by an incremental group following metacognitive training. This study assessed gains in reading performance after a meta-reading training in children 8.7 years of age diagnosed with generalized reading problems and holding incremental or entity theories of intelligence. After taking part in metacognitive training, incremental pupils, who saw their own ability as a potential they could increase, made significantly fewer errors than static pupils, who considered their skills as a gift which cannot be changed. Taken together, these two studies demonstrated how incremental theories of intelligence accounted in contributing to improve both reading components: decoding and comprehension. Moreover, support was provided to the key role of theories of intelligence in influencing reading success by increasing the students' motivation to learn and reinforcing achieved results (Faria et al., 2006).

Nevertheless, personal conceptions of intelligence seem to influence working memory (WM) training and may effect on their efficacy. The widely recognized relevance of working memory for every-day life and educational tasks is responsible for the growing attempts to implement training programs aimed at improving cognitive mechanism to maintain and manage task-relevant information during performances (Daneman and Merikle, 1996; Gathercole et al., 2004, 2006; Passolunghi, 2006; Klingberg, 2010; Loosli et al., 2011).

However, considerable evidence raised controversy concerning the efficacy of working memory training. Recently, Melby-Lervåg and Hulme (2012) conducted a systematic meta-analytic review over 23 studies to assess practical and clinical benefits as a result of working memory programs. They examined near and far-transfer effects of above-mentioned training concluding that the most consistent effects were on related but not trained in visuospatial WM memory, namely near-transfer effects (Holmes et al., 2009, 2010). Conversely, the effects on tasks going beyond the trained ability were less reliable, namely far-transfer (Morrison and Chein, 2011). Null effects of WM trainings were reported by other authors (Zinke et al., 2011). In the attempt to explain this controversy, Jaeggi et al. (2014) argued that methodological issues and individual differences could account for these contrasting results. Firstly, methodological issues concern the nature of tasks, the quality of instructions, the optimal duration and intensity of training, the adoption of group vs. single-subject research plans, the random assignment of participants to the trained and the control groups, the correct pre- and post-test evaluations, and the role of reward and motivation …Secondly, individual differences concern chronological and mental age, personality, previous abilities, as well as level of motivation. Jaeggi et al. (2014) reported the largest transfer effects only when participants showed high levels of intrinsic motivation. This was the experimental condition in which participants were not rewarded to participate or were modestly paid (Jaeggi et al., 2008, 2010). Consequently, the authors concluded that extrinsic factors, such as monetary rewards, tend to limit enjoyment and intrinsic motivation by decreasing the performance. Moreover, Jaeggi et al. (2014) found that theories of intelligence effected on benefits from WM training. In their study 175 volunteer participants were recruited and administered two working memory interventions. Results showed that individuals holding an entity theory revealed to disengage from challenging tasks and not improve following 4 weeks of intervention. Whilst, incremental participants self-reported higher levels of engagement by obtaining more consistent gains on visuospatial abilities.

## Goals

In view of these theoretical assumptions, the aim of this study is to assess the effect of personal conceptions of intelligence to strengthen the efficacy of a multidimensional intervention program to improve decoding abilities and working memory. Two 10 year-old pupils with developmental dyslexia were the participants, one holding the incremental personal conception of intelligence and one the entity conception. It was hypothesized that the multidimensional training would result in significantly more improvements in the pupil with the incremental representation of own abilities because incremental theories tend to address toward change and autonomy, to adopt more adaptive goals and efficacious strategies (Pepi et al., 2004, 2008; Jaeggi et al., 2014). The multidimensional training was implemented by integrating sessions aimed to train reading decoding abilities and sessions aimed to train verbal working memory over 3 months. More accurately, it consisted of specific tasks to improve both visual and phonological strategies such as sound blending, word segmentation, alliteration test and rhyme test, letter recognition, digraph recognition, trigraph recognition and word recognition. Tasks to enhance verbal working memory based on exercises of rapid words and non-words recognition.

This integrated intervention was chosen as a remediating intervention for our pupils because dyslexic pupils have been proven to have deficits in both phonological loop and central executive as demonstrated by their poorer performance in complex WM span tests (Jeffries and Everatt, 2004; Reiter et al., 2004; De Jong, 2006; Dahlin, 2011). Moreover, improvements in reading speed after a computerized WM training in adult dyslexic readers were documented (Horowitz-Kraus and Breznitz, 2009).

A single-subject study design was employed in this study. Such design allows primarily to evaluate the benefits of intervention programs in applied and clinical research. Moreover, it is mainly sensitive to individual differences whilst group designs are more sensitive to difference in group means.

## Background

Two Italian girls with developmental dyslexia and different personal conceptions of intelligence, attending the fifth grade of primary school, participated in the study.

Interviews with parents and teachers allowed to rebuild a picture of the learning history of the two girls. Both girls were from average socio-economic backgrounds. A history of neurological impairments or speech and language development problems were excluded. Perceptual competences concerning hearing and visual acuity were found to be typical. No family history of psychiatric diseases was reported. No emotional or behavioral disorders were reported. Both attended public primary schools and had followed conventional reading education. At age 9 both girls had been certified by a public institution as dyslexics, in line with current legislation. Families reported that their children had never been engaged in specific reading or working memory therapy.

As shown in Table 1, Alice was 10 years and 4 months old at the time of assessment, Marta was 10 years. Both girls had normal IQ (Alice: 114; Marta: 110), specific reading decoding difficulties in accuracy (Alice: 18 errors; Marta: 17 errors), and WM level was under the average (Alice: 82; Marta: 82).

**Table 1:** **Characteristics of the two participants at the Pre-Test**.

	**Alice (Incremental pupil)**	**Marta (Entity pupil)**
Chronolgical age (months)	124	120
Grade level	5th	5th
Personal conception of intelligence	Incremental	Entity
WISC-IV QIT	114 (117 standard score)	110 (112 standard score)
WISC-IV WM	82 (14 standard score)	82 (14 standard score)
WISC-IV Digit span	7 standard score	7 standard score
WISC-IV Letter-number Sequencing	7 standard score	7 standard score
WISC-IV VC	124 (42 standard score)	116 (38 standard score)
WISC-IV PR	102 (31 standard score)	100 (30 standard score)
WISC-IV PS	130 (30 standard score)	123 (28 standard score)
Reading comprehension	8 correct answers (0.18 z score)	7 correct answers (-0.27 z score)
Reading decoding accuracy	18 errors (1.95 z score)	17 errors (1.79 z score)
Reading decoding speed	2.4 syll/s (-1.1 z score)	2.37 syll/s (-1.12 z score)
Short non-word accuracy	9 errors (3.3 z score)	6 errors (1.84 z score)
Short non-word speed	37 s (0.93 z score)	36 s (0.80 z score)
Long non-word accuracy	18 errors (4.38 z score)	18 errors (4.38 z score)
Long non-word speed	59 s (0.08 z score)	90 s (2.04 z score)
Short word (high frequency of use) accuracy	6 errors (5.72 z score)	3 errors (2.49 z score)
Short word (high frequency of use) speed	31 s (2.88 z score)	28 s (2.08 z score)
Long word (high frequency of use) accuracy	8 errors (3. 3 z score)	5 errors (1.72 z score)
Long word (high frequency of use) speed	43 s (1.77 z score)	60 s (3.85 z score)
Short word (low frequency of use) accuracy	6 errors (2.41 z score)	9 errors (4.22 z score)
Short word (low frequency of use) speed	32 s (1.05 z score)	41 s (2.56 z score)
Long word (low frequency of use) accuracy	13 errors (3.28 z score)	17 errors (4.84 z score)
Long word (low frequency of use) speed	58 s (1.24 z score)	89 s (3.82 z score)

The participants were recruited on the basis of their personal conceptions of intelligence: Alice had an incremental personal conception of intelligence, while Marta had an entity profile.

Prior to beginning the study, written informed consent was provided by each participant's parents. Moreover, appropriate local ethics committee approval was obtained from the University of Palermo.

## Materials and procedure

### Procedure

The study was divided into four phases: Pre-Test phase in September, multidimensional intervention program of reading and Working memory skills from October to December, Post-Test phase in January and Follow-Up phase in March.

At the Pre-Test phase an assessment was carried out over four sessions an hour each in order to detect the baseline.The cognitive and motivational profiles of the girls were investigated. As for the cognitive profile, girls were administered a battery of reading and spelling tests commonly employed in the assessment of reading disabilities in Italy. This battery incorporated the WISC-IV (Wechsler, 2012), the Text Comprehension and Decoding Test (Cornoldi and Colpo, 2001), and the Word and Non-word Test (Zoccolotti et al., 2005). As for the motivational profile, the girls were administered the P.M.S. (Alesi et al., 2008).

### Working memory subtest derived from WISC-IV

The Wechsler Intelligence Scale for Children (WISC-IV; Wechsler, 2012) was an individual test measuring Intelligence Quotient for children with chronological age ranging 6–16. The WISC-IV provided the general Intelligence Quotient (IQ) and four indexes: Verbal Comprehension (VC), Perceptual Reasoning (PR), Working Memory (WM), and Processing Speed (PS).

The WM subtest measured the ability to monitor and manipulate mental representations and composed of two tasks: Digit Span (Forward and Backward) and Letter-Number Sequencing.

The Digit Span test consisted of sequences of numbers with increasing level of difficulty according to the length for each trial. Children were asked to repeat immediately and in the same or in the backwards order the list of numbers verbally presented at the rate of 1 number per second by the experimenter. The raw score was the number of stimuli correctly remembered.

The Letter-Number Sequencing test consisted of a series of numbers and letters. Children were asked to provide them back. The raw score was the total number of items correctly answered. Raw scores were changed into standard scores.

### Reading comprehension

The Reading Comprehension (Cornoldi and Colpo, 2001) assessed reading comprehension abilities. Pupils were asked to read a story suited to and standardized for their school grade and to answer to following 10 multiple-choice questions concerning characters and events described in the story according to their understanding of the story. The score was defined by the number of correct answers and ranged from 0 to 10. The cut-off was 5 correct choices. This cut-off (a score under 5) defines suggestions for the need of training intervention.

### Reading decoding

The Reading Decoding Test (Cornoldi and Colpo, 2001) assessed reading decoding abilities. Pupils were asked to read a text aloud. The test provided two scores: accuracy and speed. So the parameters of evaluation were the number of errors and the time of execution indicated in seconds. With regard to accuracy, the score 1 was given to errors such as long pause, addition or omission of syllables, words, or lines. The score 0.5 was given to errors such as accent shift, hesitation or self-correction. The cut-off was 8 or less errors. With regard to speed, the total score was obtained by calculating the seconds per number of syllables of text read. Average performance was score of 1.83 syllables/seconds or more.

### Phonological—visual decoding

In the Word and Non-word Reading Test (Zoccolotti et al., 2005). Three reading tasks on word and non-word reading were administered. Accuracy and speed were assessed. With regard to accuracy, for each task, the score 1 was given for each correct item and 0 for incorrect items. The speed was the time of execution indicated in seconds. The raw data thus obtained were then converted to standard scores by using tables in the manual. The cut-off was the performance below the 5th percentile.

### School motivational profiles

P.M.S. (Alesi et al., 2008) was a Multimedia Instrument, created by Visual Basic 6.0, to measure motivational factors such as the conceptions of intelligence, achievement goals, perception of controllability and causal attributions. It consisted of a story which illustrated 4 scenes from school life (1. a geography class; 2. reading a text; 3. working out a maths problem; 4. a science class) and 4 scenes from everyday life (1. assembling a jigsaw puzzle; 2. a sports race; 3. Participating in a birthday party; 4. playing a video game). Each unit presented the character (a boy/a girl) involved in school or everyday life affair and contained 4 items close-ended questions aimed at evaluating personal conceptions of intelligence (incremental vs. entity), achievement goals (learning vs. performance), controllability of effort (controllability vs. uncontrollability of effort) and causal attributions (effort, ability, luck, ease/difficulty of the task). On the whole the program provides a global qualitative index to identify Personal Conceptions of Intelligence.

Psychometric properties are as follows: regarding the validity, the factor analysis in principal components extracted two factors, the first one (incremental view) explained almost 22% of the total variance of the results and the second one (entity view) explained almost 20% of the total variance of the results in normative sample. The test-retest reliability ranged from 0.41 to 0.79 (Alesi and Pepi, 2008).

### After the pre-test phase the girls took part in T.I.R.D.

Multimedia Training for the Rehabilitation of Dyslexia (Rappo and Pepi, 2010) and in a Multimedia Training to improve the WM abilities (Sacchi, 2012)^1^. The treatment program took place over 12 sessions twice a week in a quiet room, one pupil at a time, with the same experimenter providing training tasks (See Table 2).

**Table 2:** **Treatment program (T.I.R.D. and Multimedia Training to improve the WM abilities) daily sessions**.

	**Tasks of the T.I.R.D**.	**Training of WM (number of cards)**
Day 1	Fusion and alliteration	Memory task: 4/6 cards
Day 2	Segmentation and rhymes	Memory task: 6/8 cards
Day 3	Fusion and alliteration	Memory task: 6/8 cards
Day 4	Segmentation and rhymes	Memory task: 8/12 cards
Day 5	Letter search and digraphs search	Memory task: 8/12 cards
Day 6	Letter search and trigraph search	Memory task: 12/16 cards
Day 7	Fusion and alliteration	Memory task: 12/16 cards
Day 8	Segmentation and rhymes,	Memory task: 16/20 cards
Day 9	Fusion and alliteration	Memory task: 16/20 cards
Day 10	Segmentation and rhymes,	Memory task: 20/24 cards
Day 11	Word search and reading of words written in unusual format	Memory task: 20/24 cards
Day 12	Word search and reading of words written in unusual format	

The T.I.R.D. consisted in specific tasks to rehabilitate both visual and phonological strategies. The software, written in the coding language Visual Basic 6.0 (Perry, 1998), has three parts, two for collecting administrative data and one for training. The first administrative part gathers information about the testing situation and participants' demographics. The second administrative part is a summary of all the collected data. The training program consisted in 356 growing difficulty tasks and subdivided into four units. Units 1 and 3 included phonological tests such as fusion, segmentation, alliteration, rhymes, and non-word reading. Units 2 and 4 included visual tests such as letter search, digraphs search, trigraph search, word search, and reading of words written in unusual format. The third part of the software consisted of the data visualization form containing the results of the training. The training was carried out over 12 sessions, 40 min long.

The units of T.I.R.D. were reinforced by specific units to improve verbal working memory.

**Figure 1:**
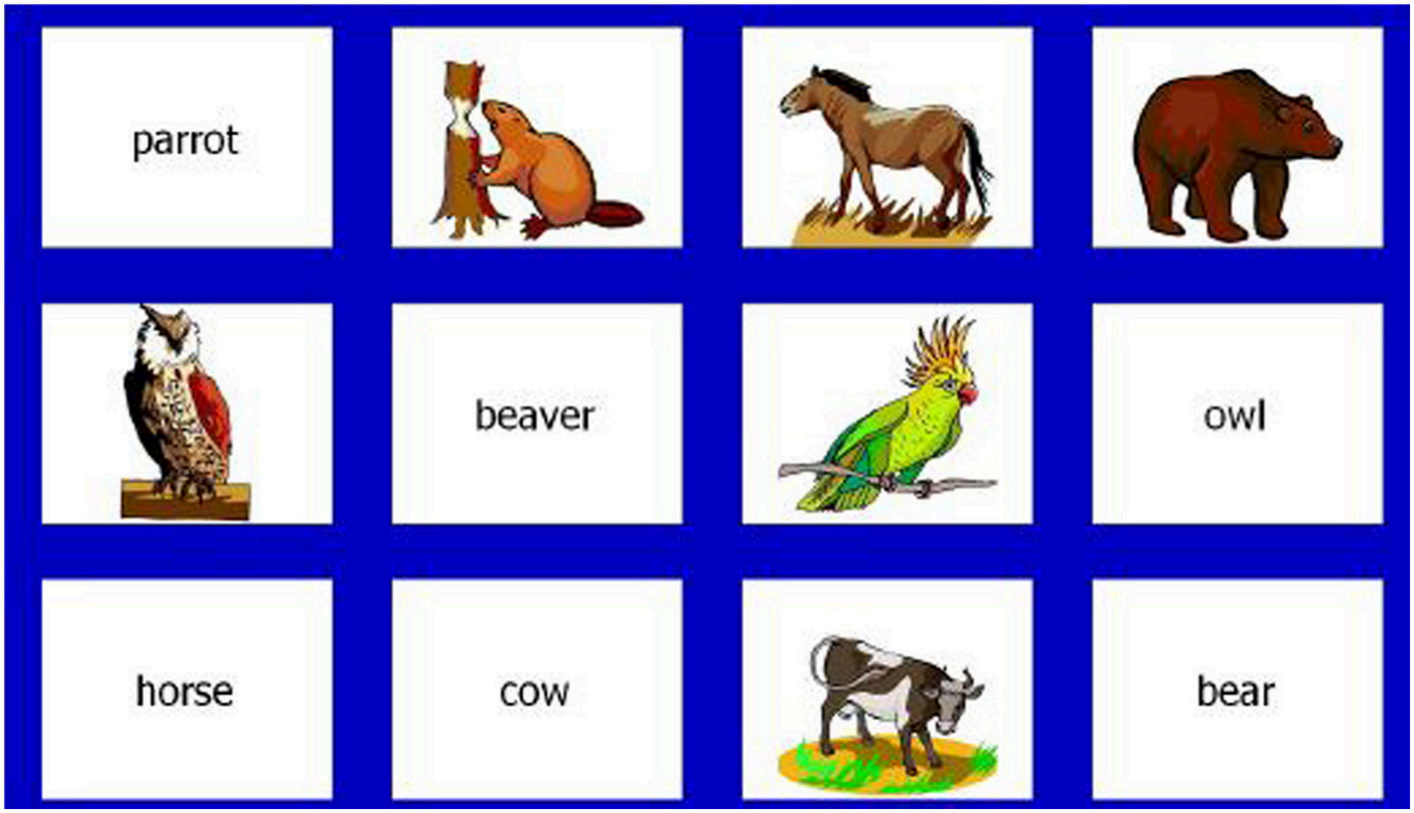
**Example of Working Memory training—second and third day of the program**.

This free Software was produced by Ivana Sacchi (www.ivana.it). The training consisted in memory tasks with a one to one match activities of the cells. Cards containing animal images and animal names appeared on the display and the task was to match an image to the corresponding name (see Figure 1). The number of cards to be used varied from 4 to 30. The software allowed the creation of specific fully customized routes: the color of the graphic interface could be changed, cards with new designs on them could be inserted, the font (printed capital letters or italics) could be changed and a sound reinforcement (the selection of the card, exact pair, couple wrong) could be added. Correct image—name pairs disappeared, after having been matched. The exposure time of the cards could be set at the top of the task (the time required to study the position of the cards and hold them in mind).The WM training was carried out in 11th sessions of increasing difficulty which lasted no more than 10 min each and was administered after the software TIRD. More specifically, the first session allowed girls to familiarize with the software with 4/6 cards. On the first day children were familiarized with the task.

On the second and third day the number of tiles was 6/8 and cards covered after 5 s. On the fourth and 5th day the number of tiles was 8/12 and cards covered after 10 s. On the 6th and 7th day the number of tiles was 12/16 and cards covered after 15 s. On the 8th and 9th day the number of tiles was 16/20 and cards covered after 20 s. On the 10th and 11th day the number of tiles was 20/24 and cards covered after 25 s.

Participants were asked to correctly match a figure and a corresponding word. Each pair correctly operated by the child was reinforced with a sound, while each incorrect pair was not reinforced. The use of this software had been adapted in order to strengthen the manipulation of data hold in the mind. After the matching activity, when image—name pairs disappeared, the girls had to repeat the name of animals beginning with the letter named by the psychologist. For example, in the second and third day of the program (Figure 1), the girls were asked to remember the names of animals beginning with “b,” such as beaver and bear. During each session the pupils could train for up to 10 min.

We selected this program to train WM because it fitted well with the nature of tasks provided by the TIRD and because each unit would not be too long (lasting no more than 50 min), and tiring or boring for the girls.

Following the multidimensional intervention program phase, the post-test phase included re-evaluation of reading decoding difficulties, in both accuracy and speed, and the Working Memory IQ. The 3-month follow-up consisted of the same tasks as at post-test.

## Results

The Reliable Change Index^2^ was used to verify changes between Pre-Test, post-test, and follow-up (Jacobson and Truax, 1991).

After the multidimensional intervention program phase both Alice and Marta improved their accuracy of reading decoding accuracy from Pre-Test to post-test. In particular Alice decreased the number of the errors from 18 to 14, while Marta from 17 to 13 (*SD* = 6.2; reliability = 0.95). None of the two significantly improved in reading speed. Regarding the WM, only Alice showed significant improvements. In particular, the IQ scores improved from 82 to 100 (*SD* = 13.4; reliability = 0.88). Alice and Marta did not show any change from Post-Test to Follow-Up.

Finally, only Alice showed significant improvements from Pre-Test to Follow-Up in reading decoding accuracy and WM abilities. In particular Alice decreased the number of errors from 18 to 12.5, and improved her WM ability from 82 to 103 (See Tables 3–6).

**Table 3:** **Characteristics of Alice in the Pre-Test, Post-Test, and Follw-up**.

	**Pre-test**	**Post-test**	**Follw-up**
WISC-IV QIT	114 (117 standard score)	124 (119 standard score)	126 (121 standard score)
WISC-IV WM	82 (14 standard score)	100 (20 standard score)	103 (21 standard score)
WISC-IV Digit Span	7 standard score	8 standard score	9 standard score
WISC-IV Letter-Number Sequencing	7 standard score	12 standard score	12 standard score
Reading decoding accuracy	18 errors (1.95 z score)	14 errors (1.31 z score)	12.5 errors (1.06 z score)
Reading decoding speed	2.4 syll/s (-1.1 z score)	2.37 syll/s (-1.12 z score)	2.51 syll/s (-1.01 z score)
Short non-word accuracy	9 errors (3.3 z score)	10 errors (3.79 z score)	7 errors (2.33 z score)
Short non-word speed	37 s (0.93 z score)	53 s (2.95 z score)	45 s (1.94 z score)
Long non-word accuracy	18 errors (4.38 z score)	13 errors (2.62 z score)	11 errors (1.92 z score)
Long non-word speed	59 s (0.08 z score)	66 s (0.52 z score)	60 s (0.14 z score)
Short word (high frequency of use) accuracy	6 errors (5.72 z score)	5 errors (4.65 z score)	3 errors (2.49 z score)
Short word (high frequency of use) speed	31 s (2.88 z score)	27 s (1.81 z score)	24 s (1.01 z score)
Long word (high frequency of use) accuracy	8 errors (3. 3 z score)	6 errors (2.25 z score)	4 errors (1.19 z score)
Long word (high frequency of use) speed	43 s (1.77 z score)	44 s (1.89 z score)	41 s (1.52 z score)
Short word (low frequency of use) accuracy	6 errors (2.41 z score)	7 errors (3.1 z score)	5 errors (1.81 z score)
Short word (low frequency of use) speed	32 s (1.05 z score)	37 s (1.89 z score)	32 s (1.05 z score)
Long word (low frequency of use) accuracy	13 errors (3.28 z score)	12 errors (2.87 z score)	6 errors (0.54 z score)
Long word (low frequency of use) speed	58 s (1.24 z score)	54 s (0.89 z score)	46 s (0.19 z score)

**Table 4:** **Characteristics of Marta in the Pre-Test, Post-Test, and Follw-up**.

	**Pre-test**	**Post-test**	**Follw-up**
WISC-IV QIT	114 (117 standard score)	110 (108 standard score)	109 (107 standard score)
WISC-IV WM	82 (14 standard score)	85 (15 standard score)	82 (14 standard score)
WISC-IV Digit Span	7 standard score	7 standard score	7 standard score
WISC-IV Letter-Number Sequencing	7 standard score	8 standard score	7 standard score
Reading decoding accuracy	17 errors (1.79 z score)	13 errors (1.15 z score)	18 errors (1.95 z score)
Reading decoding speed	2.37 syll/s (-1.12 z score)	2.37 syll/s (-1.12 z score)	2.18 syll/s (-1.27 z score)
Short non-word accuracy	6 errors (1.84 z score)	9 errors (3.3 z score)	10 errors (3.79 z score)
Short non-word speed	36 s (0.80 z score)	40 s (1.31 z score)	47 s (2.19 z score)
Long non-word accuracy	18 errors (4.38 z score)	9 errors (1.21 z score)	14 errors (2.97 z score)
Long non-word speed	90 s (2.04 z score)	73 s (0.97 z score)	82 s (1.54 z score)
Short word (high frequency of use) accuracy	3 errors (2.49 z score)	1 error (0.34 z score)	2.5 errors (1.96 z score)
Short word (high frequency of use) speed	28 s (2.08 z score)	31 s (2.88 z score)	27 s (1.81 z score)
Long word (high frequency of use) accuracy	5 errors (1.72 z score)	3 errors (0.67 z score)	2 errors (0.14 z score)
Long word (high frequency of use) speed	60 s (3.85 z score)	48 s (2.38 z score)	52 s (2.87 z score)
Short word (low frequency of use) accuracy	9 errors (4.22 z score)	10 errors (4.82 z score)	9 errors (4.22 z score)
Short word (low frequency of use) speed	41 s (2.56 z score)	33 s (1.22 z score)	43 s (2.9 z score)
Long word (low frequency of use) accuracy	17 errors (4.84 z score)	9 errors (1.71 z score)	11 errors (2.5 z score)
Long word (low frequency of use) speed	89 s (3.82 z score)	77 s (2.90 z score)	82 s (3.34 z score)

**Table 5:** **Reliable change index for alice**.

	**Pre-test to Post-test**	**Post-test to Follow-up**	**Pre-test to Follow-up**
Reading decoding accuracy	RCI > 1.96; *p* < 0.05	Not significant	RCI > 1.96; *p* < 0.05
Reading decoding speed	Not significant	Not significant	Not significant
WM	RCI > 1.96; *p* < 0.05	Not significant	RCI > 1.96; *p* < 0.05

**Table 6:** **Reliable change index for marta**.

	**Pre-test to Post-test**	**Post-test to Follow-up**	**Pre-test to Follow-up**
Reading decoding accuracy	RCI > 1.96; *p* < 0.05	Not significant	Not significant
Reading decoding speed	Not significant	Not significant	Not significant
WM	Not significant	Not significant	Not significant

**Figure 2:**
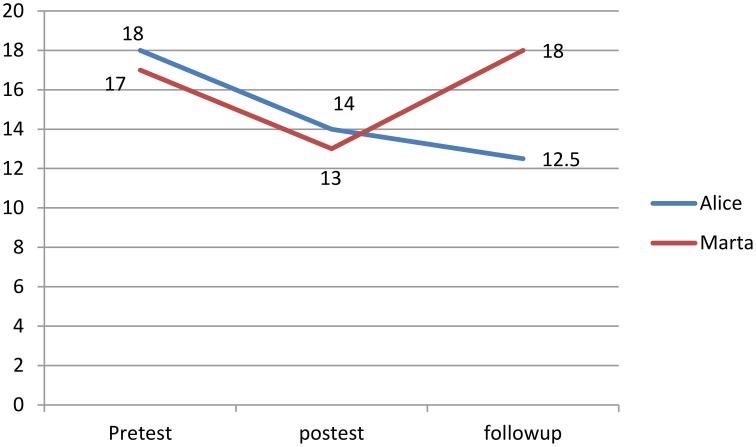
**Reading decoding accuracy scores (errors)**.

**Figure 3:**
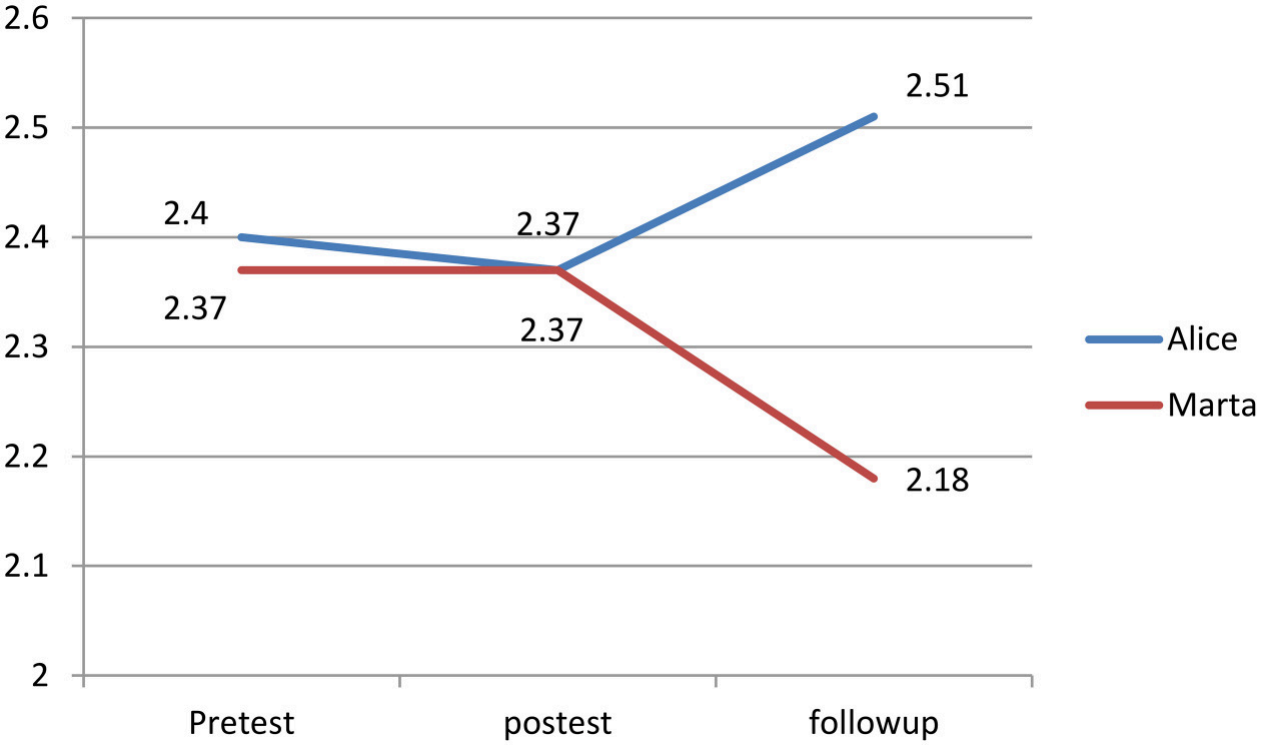
**Reading decoding speed (syllables/seconds)**.

**Figure 4:**
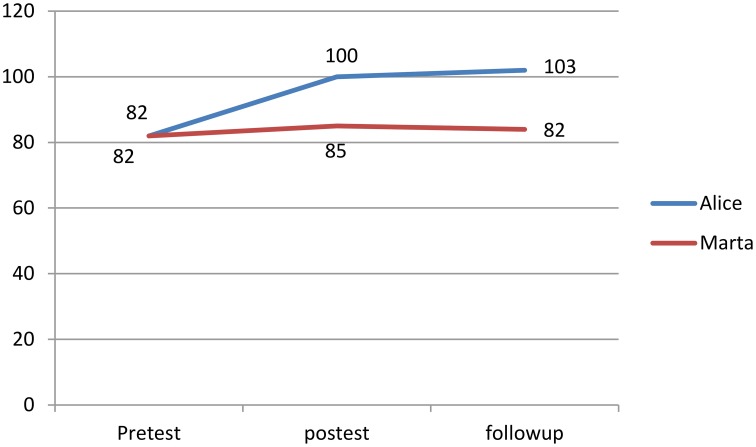
**WM standard scores**.

In contrast with Marta, Alice's scores, at Pre-Test, post-test and follow-up phases, showed a steady improvement in reading decoding accuracy and WM ability (See Figures 2–4).

## Discussion

The goal of this study was to assess the role of personal conceptions of intelligence in order to strengthen the efficacy of a multidimensional intervention program to improve decoding abilities and working memory in dyslexia. In order to do this, we described two case studies of two Italian pupils with developmental dyslexia and low WM abilities. Alice had an incremental personal conception of intelligence, whilst Marta showed an entity personal conception of intelligence. Their performances on reading decoding and WM abilities were equivalent at baseline and were compared from Pre-Test to post-test and follow up. Alice was 10 years and 4 months old at the time of testing, Marta was 10 years. Both the girls had normal IQ. They had specific reading decoding difficulties in accuracy and speed and their WM abilities were under the average. Both Alice and Marta were administered a multidimensional training which combined units aimed at training reading decoding abilities and units aimed to train verbal working memory over 3 months. Following the training, with concern regards to reading accuracy abilities, both pupils seem to have enhanced their skills. The performance on reading accuracy tasks improved because errors decreased of 22.22% in Alice and 23.53% in Marta.

Concerning working memory abilities, Alice improved more than Marta. The performance on WM tasks significantly increased by 42.86% in Alice and only 7.14% in Marta.

As previously described, the two girls showed similar cognitive profiles as regards to their performance on WISC Working Memory (Marta = 14; Alice = 14) and WISC Perceptual Reasoning (Marta = 31 and Alice = 30). Differences were found on WISC Verbal Comprehension (Marta = 42; Alice = 38) and WISC Processing Speed (Marta = 30; Alice = 28), but these differences cannot be considered significant (Wechsler, 2012). It may be possible that processing abilities create a better condition to benefit from a training were based on memory tasks requiring to match an animal image to the corresponding name. Another possible explanation could be the influence of the T.I.R.D. Multimedia Training for the Rehabilitation of Dyslexia consisting in specific tasks to rehabilitate both visual and phonological strategies. This is consistent with more consistent and stable improvement in reading decoding accuracy shown by Marta.

However, given that the intellectual profiles between the two girls were equivalent, it was hypothesized that the main factor contributing for differences in training gains could be Alice's personal conceptions of intelligence. In particular her incremental personal conception of intelligence could act as a potential mechanism of change by orienting her perception of abilities as something changeable and improvable through effort and hard work. Moreover, this is consistent with previous studies (Pepi et al., 2004, 2008) and supports an important role for conceptions of intelligence in influencing school success, both in terms of the students' willingness to learn and of the achieved results (Faria et al., 2006).

The most interesting result was that significant differences between Alice and Marta were maintained at a 3-month follow-up. In Alice, changes of both accuracy and working memory were more consistent at Follow-up: advantages were maintained after 3 months. In particular, the performance on reading accuracy tasks had improved and errors decreased of 30.56%. Her performance on WISC-IV WM increased by 50% from Pre-Test to Follow-up showing a gain from post-test to follow-up of 5%. Contrastingly, in Marta the significant improvement in accuracy reading decoding from Pre-Test to Post-test disappeared at Follow-up.This result was necessary evidence not only for the efficacy of the multidimensional intervention proposed, but for the role of personal conceptions of intelligence in effecting improvements as well as maintaining positive effects. This is a very exciting result as it supports the ongoing debate concerning the maintenance over time of WM training programs (Melby-Lervåg and Hulme, 2012) by providing evidence of a potential mechanism able to effect reliable long-term improvements.

On the whole, the pupil who believes that it is possible to enhance one's abilities and performance will tend to interpret and manage learning as a long-term process. This means “…. to defer gratification, foregoing chances to succeed on difficult tasks in the immediate future. Such students prefer learning goals based on their desire to acquire new knowledge and master new skills” (Alesi et al., 2012, p. 971). In contrast, the static pupil who believes that abilities are relatively fixed will tend to focus mostly on current performance because she interprets the effort as an indicator of her inadequate ability. Consequently, she prefers easy tasks and employs superficial strategies in order to favor easily achievable goals which ensure positive judgements of own capacity (Pepi et al., 2000). As such, personal conceptions of intelligence would be a good prognostic factor in the evaluation of programs aimed at overcoming specific deficits in decoding or working memory domains. The way in which intelligence is conceived supports the readiness to surmount specific difficulties through treatment programs because students are more likely to be oriented toward change and autonomy, adopt more successful strategies and process decisions and action plans with ever increasing awareness. The incremental conceptions of intelligence predict an upward evolutionary trajectory in training programs, whilst the entity conceptions predict a flat trajectory (Dweck, 1999).

## Concluding remarks

The main strength of this study lies in contributing to the current literature with respect to the debate around the controversy concerning the efficacy of domain-specific intervention programs. The findings further support the relevance of treatment programs in which both specificity of deficits and individual differences are taken into account (Jaeggi et al., 2014). However, the data needs to be interpreted with caution because it derived from the analysis of two case studies weakening the generalizability of the current findings. As suggested by the hierarchy of evidence proposed by Sackett (1989) this research, ranked as case report, shows a low level of evidence (V level) which “may contain extremely useful information about clinical course and prognosis, but can only hint at efficacy” (pag.3S). Moreover, another possible shortcoming of this study is that at the present time we have data derived from follow-up at 3 months. Long-term maintenance of obtained gains needs to be re-evaluated by follow-up at 6 months. Finally, the direction of the link between motivational patterns and domain-specific impairments is theoretically unclear and is now the subject of wide discussion. It is more correct to suppose a mutual relationship in which maladaptive motivational patterns are a consequence of reading or WM deficit although reading and WM deficits may also lead to more negative motivational profiles.

Notwithstanding these limitations, the research carried out suggests some interesting implications on the educational and clinical fields for future practice. This can be used to develop targeted evidence-based programs which need to take account both of the specificity of disability and of the factors relating to motivational domain in order to maximize the maintenance and generalization of obtained improvements.

### Conflict of interest statement

The authors declare that the research was conducted in the absence of any commercial or financial relationships that could be construed as a potential conflict of interest. The reviewer Barbara Carretti and handling Editor declared a current collaboration and the handling Editor states that the process nevertheless met the standards of a fair and objective review.

## Footnotes

^1^Free Educational Software www.ivana.it.

^2^The formula for the Reliable Change Index is: RC = (X_2_− X_1_)/S_diff_, where X_2_− X_1_ is the difference between posttest and pretest scores and S_diff_ is the standard error of measurement (www.abdn.ac.uk/j.crawford/pages/dept/Compare_Two_Cases.htm).
